# Emodin affects ERCC1 expression in breast cancer cells

**DOI:** 10.1186/1479-5876-10-S1-S7

**Published:** 2012-09-19

**Authors:** Jian-min Fu, Jie Zhou, Jian Shi, Jian-sheng Xie, Li Huang, Adrian YS Yip, Wings TY Loo, Louis WC Chow, Elizabeth LY Ng

**Affiliations:** 1 Department of Breast Disease, Shenzhen Maternity and Child Healthcare Hospital, Southern Medical University, Shenzhen, China; 2 Organisation for Oncology and Translational Research, Hong Kong and UNIMED Medical Institute, Hong Kong SAR

## Abstract

**Background:**

Multi-drug resistance to chemotherapeutic agents is a major cause of treatment failure in breast cancer. In this study, we investigated the effects of emodin on reversing the multi-drug resistance, examined the ERCC1 protein expression in breast cancer cell line, and explored the relationship between reversal of multi-drug resistance and ERCC1 protein expression.

**Methods:**

MTT assay was conducted to test the cytotoxicity of adriamycin and cisplatin to MCF-7/Adr cells with and without emodin pretreatment, and Western blot was performed to examine the ERCC1 protein expression.

**Results:**

MCF-7/Adr cells had 21-fold and 11-fold baseline resistances to adriamycin and cisplatin, respectively. When emodin was added to the cell culture at the concentration of 10 μg/ml, the drug resistance was reduced from 21 folds to 2.86 folds for adriamycin, and from 11 folds to 1.79 folds for cisplatin. MCF-7/Adr cells treated with two concentrations (10μg/mL and 20μg/mL) of emodin, after 2, 4, 6, 10 days, the trend of ERCC1 expression was gradually decreased and the reduction was more obvious comparatively at the concentration of 20μg/mL.

**Conclusions:**

Emodin could reverse the multi-drug resistance in MCF-7/Adr cells and down-regulate ERCC1 protein expression.

## Background

Excision repair cross complementation group 1 (ERCC1) protein encoded by *ERCC1* gene is a key player in nucleotide excision repair (NER), and our previous work and others have demonstrated that this protein is also expressed in breast cancer [1,2]. The NER system represented by ERCC1 is extensively involved in human cell DNA repair after damage, but over-expression of ERCC1 can lead to multi-drug resistance to chemotherapy in cancer treatment [3,4].

In recent years, the expression of ERCC1 was extensively studied in endometrial cancer [5], ovarian cancer [6], non-small cell lung cancer [7-9], nasopharyngeal cancer [10] and thymic cancer [11]. It was deemed to predict response to anti-cancer treatment and possibly have a prognostic role. According to the latest systematic review of predictive value of multidrug resistance-associated proteins (MDR1, MRP1, MRP2 and MVP), topoisomerase II and ERCC1, ERCC1 was a promising predictive marker for survival in some patients [12]. However, the study of ERCC1 in breast cancer is limited. Kim [13] found that ERCC1 expression is low in triple-negative breast cancer subtypes, but the relationship with survival is still unknown. ERCC1 might have its roles in DNA repair systems in breast cancer, but its contribution to drug resistance remains unclear.

Emodin (EMD) is a natural anthraquinone compound extracted from the rhizome of rhubarb. The traditional Chinese medicinal herb was widely used for treatment of various ailments and the anti-cancer activity of EMD was demonstrated in some studies [14-16]. The ability to reverse the multi-drug resistance to cancer chemotherapeutic agents was also shown in previous pharmacological studies [17,18]. In this *in vitro* study, multi-drug resistant breast cancer cell line MCF-7/Adr was exposed to different levels of EMD. Drug sensitivity and ERCC1 expression were studied, so as to explore the role of ERCC1 in breast cancer multi-drug resistance and the effect of EMD on reversing such resistance.

## Methods

### Drugs and reagents

Adriamycin (ADM, Haizheng Pharmaceuticals Co., Ltd, Zhejiang, China.), cisplation (DDP, Nanjing Pharmaceuticals Co., Ltd, Jiangsu, China), Emodin (EMD, China National Institute for the Control of Pharmaceutical and Biological Products, Beijing China), cell culture medium RPMI-1640 (GIBCO,USA), methyl thiazolyl tetrazolium (MTT) assay kit (Sigma,USA), mouse anti-human ERCC1 monoclonal antibody (Santa Cruz,USA), horseradish peroxidase-labeled goat anti-mouse secondary antibody (Sigma,USA) were all commercially obtained. ADM and EMD were reconstituted with sterile injection water to make 2 mg/ml and 20 mg/ml stock solutions, respectively; and DDP was reconstituted with normal saline solution to make 5 mg/dl stock solution. All the stock solutions were divided in appropriate aliquots and kept in 4°C refrigerator. Application solutions were made immediately before use by adding culture medium.

### Cell lines

Multi-drug resistant breast cancer cell line MCF-7/Adr and its drug sensitive parent cell line MCF-7 were obtained from Guangzhou DaHui Biotech Co., Ltd. China. The cells were cultured in RPMI-1640 medium supplemented with 10% fetal bovine serum, penicillin 100 U/ml and streptomycin 100 μg/m1, in 5% CO_2_, saturated humidity, 37°C incubator. The cells show adherent growth. In vitro study was conducted when the cells reach logarithmic growth phase.

### MTT assay

Cells at logarithmic growth phase were seeded on 96-well culture plates, with 6×10^3^ cells in each well. After 24 hours of culture, the cells were evenly attached to the bottom of the plate. ADM, DDP and EMD of 5 concentration gradients were added. After 72 hours, the medium was removed, 100 μl of MTT reagent (5 mg/ml) was added to each well, and the plate was cultured for another 4 hours. Then the MTT solution was removed, 200 μl of dimethyl sulphoxide (DMSO) was added. The plate was shaken for 15 minutes to fully dissolve the MTT. Absorbance in each well was determined at 490 nm with enzyme-linked immunoassay detector. Cell viability was determined according to the follow equation: Cell survival rate = (absorbance in the drug test group/absorbance in control group)×100%.

### Calculation and statistical analysis

The 50% lethal concentration (IC_50_) was calculated according to Reed-Muench formula and expressed as mean ± SD. The drug resistance = (IC_50_ of MCF-7/Adr) ÷ (IC_50_ of MCF-7). Drug resistance reversal = (IC_50_ of MCF-7/Adr cultured without EMD) ÷ (IC_50_ of MCF-7/Adr cultured with EMD).

### ERCC1 expression by Western blotting

MCF-7 and MCF-7/Adr cells at logarithmic growth phase were seeded into 6-well plate with 1.2×10^4^ cells in each well. MCF-7 cells in the first well were used as negative control. MCF-7/Adr cells in the second well were used as blank control. MCF-7/Adr cells in the other 4 wells were exposed to 10 μg/ml and 20 μg/ml of EMD. After cell culture for 2, 4, 6 and 10 days, the cells were harvested, and total cell protein was extracted after cell-lysis. The cell protein was subjected to sodium dodecyl sulphate polyacrylamide gel electrophoresis (SDS-PAGE). The separated proteins were transferred to nitrocellulose membrane. After blocking in 5% skim milk for 1.5 hours, the membrane was incubated with mouse anti-human ERCC1 monoclonal antibody overnight, and then horseradish peroxidase-labeled goat anti-mouse secondary antibody for 2 hours. After washing, the film was developed in darkroom per protocol.

## Results

**Figure 1:**
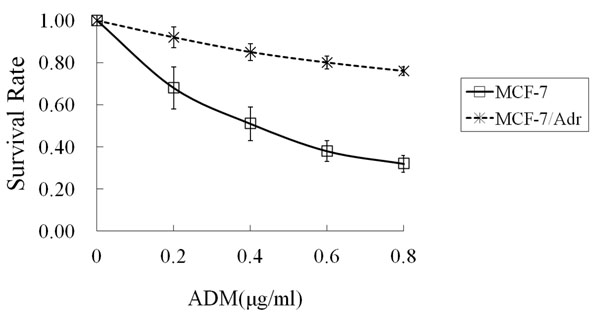
**The survival rate of MCF-7 and MCF-7/Adr cells treated with ADM for 72 hours** Abbreviation: ADM: Adriamycin. Data shown are mean ± SD from quadruplicate determinations. The experiment was performed three times with similar results.

**Figure 2:**
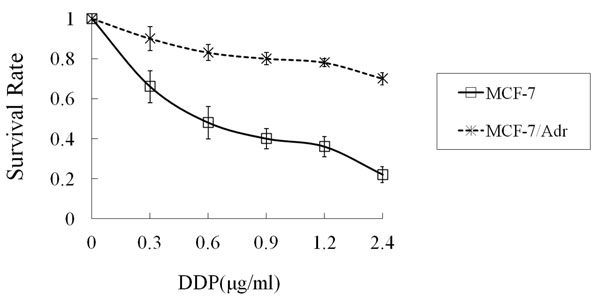
**The survival rate of MCF-7 and MCF-7/Adr cells treated with DDP for 72 hours** Abbreviation: DDP: Cisplatin. Data shown are mean ± SD from quadruplicate determinations. The experiment was performed three times with similar results

**Figure 3:**
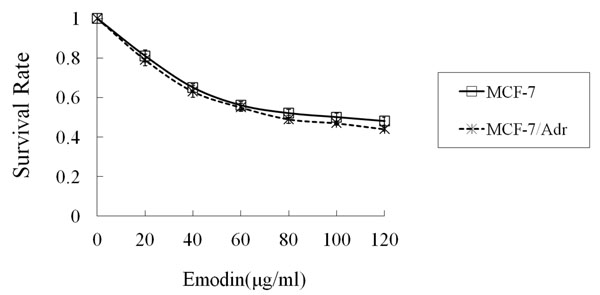
**The survival rate of MCF-7 and MCF-7/Adr cells treated with emodin for 72 hours** Data shown are mean ± SD from quadruplicate determinations. The experiment was performed three times with similar results.

When MCF-7 and MCF-7/Adr cells were exposed to ADM gradient solutions, the IC_50_ of MCF-7/Adr was 21 times higher than MCF-7 (8.75 ± 0.39 μg/ml *vs.* 0.41 ± 0.11 μg/ml, *P* < 0.01) (Figure 1). When MCF-7 and MCF-7/Adr cells were exposed to DDP gradient solutions, the IC_50_ of MCF-7/Adr was 11 times higher than MCF-7 (6.34 ± 0.32 μg/ml *vs.* 0.56 ± 0.13 μg/ml, *P*<0.01) (Figure 2). After exposure to EMD at various concentrations, the IC_50_ was 100 ± 0.35 μg/ml for MCF-7 and 79 ± 0.28 μg/ml for MCF-7/Adr (*P* > 0.05) (Figure 3).

**Figure 4:**
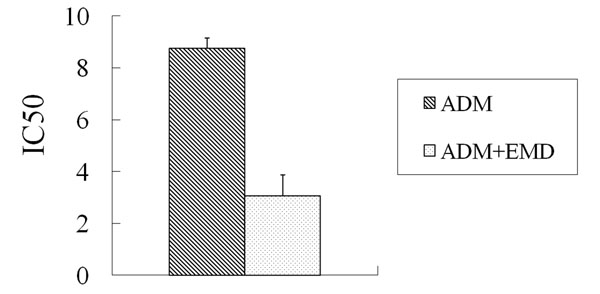
**The IC50 of ADM with and without emodin for MCF-F/Adr cells** ADM: Adriamycin

**Figure 5:**
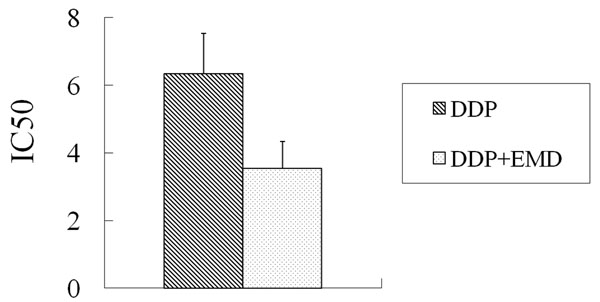
**The IC50 of DDP with and without emodin for MCF-7/Adr cells** DDP: Cisplatin

For MCF-7/Adr cells, the ADM IC_50_ was reduced from 8.75 ± 0.39 μg/ml without EMD treatment to 3.06 ± 0.26 μg/ml after treatment with 10 μg/ml EMD (*P* < 0.01). EMD had a 2.86-fold reversal of ADM IC_50_ for MCF-7/Adr cells (Figure 4). For MCF-7/Adr cells, the DDP IC_50_ was reduced from 6.34 ± 0.32 μg/ml without EMD treatment to 3.54 ± 0.28 μg/ml after treatment with 10 μg/ml EMD (*P* < 0.05). EMD had a 1.79-fold reversal of ADM IC_50_ for MCF-7/Adr cells (Figure 5). However, the change in IC_50_ of both ADM and DDP was not statistically significant after treatment with EMD.

**Figure 6:**
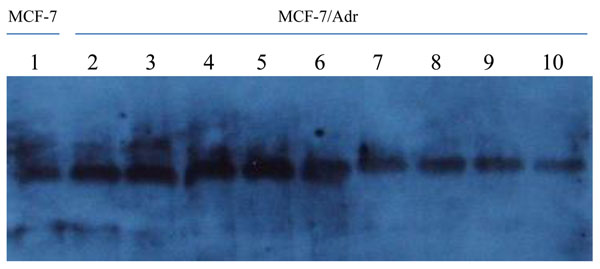
**Western blotting results** Lane 1: Negative/loading control; lane 2: Blank control; lane 3, 5, 7, 9: Cells exposed to 10μg/ml EMD; lane 4, 6, 8, 10: Cells exposed to 20μg/ml EMD

The baseline ERCC1 expression was higher in MCF-7/Adr cells than MCF-7 cells. When MCF-7/Adr cells were treated with EMD treatment at 10 μg/ml for 2, 4, 6 and 10 days, ERCC1 expression progressively decreased. Significantly greater inhibition of ERCC1 expression was evident when the cells were exposed to 20 μg/ml of EMD for 2, 4, 6 and 10 days. At each time point, EMD at 20 μg/ml shows a higher inhibitiory effect (Figure 6).

Baseline ERCC1 expression was lower in MCF-7 (negative control, Lane 1) than in MCF-7/Adr (blank control, Lane 2). When MCF-7/Adr cells were exposed to EMD (10μg/ml) for 2 (Lane 3), 5 (Lane 5), 6 (Lane 7) and 10 (Lane 9) days, ERCC1 expression was progressively inhibited. When MCF-7/Adr cells were exposed to 20 μg/ml of EMD, significantly greater inhibition was also observed at each time point (Lanes 4, 6, 8 and 10).

## Discussion

Multi-drug resistance to chemotherapeutic agents is a major cause of treatment failure in breast cancer, because chemotherapy plays ever increasing role in the systemic treatment modalities of breast cancer both in neoadjuvant and adjuvant chemotherapy [19,20]. For patients who cannot obtain clinical remission during adjuvant chemotherapy and those with recurrence and metastasis after initial treatment, reversal of multi-drug resistance is of crucial importance for improving clinical outcome [21,22]. After resection of the primary tumor, there is no validated clinical parameter to predict the sensitivity to chemotherapy. Therefore, it is essential to explore the molecular mechanisms of multi-drug resistance and the reversal strategy are very important in breast cancer treatment [23-26].One of the mechanisms of tumor resistance to cisplatin is increased NER activity, in particular increased levels of *ERCC1*, which is a key gene involved in NER of damaged DNA by ultraviolet radiation or chemical agents. The 5’ incision made by the ERCC1-XPF complex was deemed as a rate-limiting step in the NER pathway, as shown by an increase in excision activity in extracts from non-cisplatin resistant cells after addition of purified ERCC1-XPF protein, compared with no increase in excision activity after addition of ERCC1-XPF to extracts from cisplatin-resistant cells [27,28]. The potential use of ERCC1 mRNA expression as a predictive marker for the effectiveness of cisplatin-based chemotherapy is an important area of clinical research [29].

Patients with lower DNA repair capacity are more chemosensitive than those who carry a proficient DNA repair system. In early, it was shown that elevated DNA repair capacity is associated with drug resistance in lung cancer cell lines [30], and it was suggested that modulation of DNA repair mechanisms, such as the incorporation of specific DNA repair inhibitors in therapeutic regimens. Although low expression of ERCC1 is related to carcinogenesis, high expression could enhance the NER, leading to rapid repair of the damaged tumor DNA after chemotherapy, a plausible mechanism of multi-drug resistance in many cancers [31]. Thus, we processed a serial studies about ERCC1 in breast cancer and look for novel anti-cancer strategies to avoid drug resistance and improve treatment outcomes.

EMD is a chemical compound of the anthraquinone family mainly derived from the root of *Rheum palmatum*, a widely used herb in traditional Chinese medicine, with a variety of anti-bacteria, anti-tumor and anti-constipation properties. Studies at the cellular level have shown that EMD affects cell proliferation by inhibiting DNA synthesis, prolonging the cell cycle duration, and suppressing mitosis. In our study, when the two breast cancer cells were exposed to 0~120 μg/ml of EMD, the IC50 was 100 ± 0.35 μg/ml for MCF-7 and 79 ± 0.28 μg/ml for MCF-7/Adr. At 20μg/ml EMD, cell viability was over 85% for both MCF-7 and MCF-7/Adr. Therefore, we selected 10μg/ml EMD at which no cell growth inhibition was observed as the starting dose for the reversal test. Our results showed that in MCF-7/Adr cells, EMD could reduce the ADM-resistance by 2.86 folds, and the DDP resistance was reduced by 1.79 folds. These results confirm that EMD is a highly effective drug-resistance reversal agent with low toxicity.

High expression of NER repairing ability enhancement is considered as one of the mechanisms of tumor drug resistance [32,33]. In this study, two concentrations of emodin, 20μg/mL and 10μg/mL, were used to deal with low toxicity concentration MCF-7/Adr cell line, in 2, 4, 6 and10 days and the expression of ERCC1 protein was examined by Western blotting. The results showed higher levels of ERCC1 protein expression in MCF-7/Adr cell lines than in MCF-7 ones, but after emodin treatment, the ERCC1 protein levels decreased further and is significantly time-dependent and possibly dose-dependent as greater inhibitory effect was observed in the concentration of 20μg/ml. Given that only two concentration were studied, it may still have a certain concentration range to explore further the true dose-effect relationship.

Many new anti-cancer agents is being developed in recent years, but many studies have now focused on the reversal of chemoresistance in tumor in order to maximize the treatment capacity of existing chemotherapeutic agents. Some medicinal herbs may hold great potential in this field. This *in vitro* study found that EMD can significantly reverse the multi-drug resistance and reduce ERCC1 expression in breast cancer cells. Further studies are warranted to explore how ERCC1 plays role in reversing drug resistance and more pre-clinical evidence is necessary before the use emodin will be studied in clinical trials.

## Conclusions

In this *in vitro* study, we noticed that ERCC1 expression might be associated with drug resistance and that emodin might play a possible role of reversing drug resistance. This is a preliminary finding which needs further investigation to explore the use of emodin in breast cancer treatment. However, we must emphasize that with recent advances of molecular biology, breast cancer was categorized into many different molecular subtypes which might possess different characteristics from carcinogenesis to metastasis. Therefore, the study of a single biomarker might be further complicated. Nevertheless, we believe further studies on ERCC1 expression in breast cancer and emodin in facilitating the reversal of drug resistance are warranted.

## Competing interests

The author(s) declare that they have no competing interests.

## Authors' contributions

JF, JZ, JS, JX and LH carried out the pre-clinical research. JF, AY, WL and LC participated in writing the manuscript. WL performed statistical analysis. JF and LC provided expert opinion for the study. JF was the initiator of the study.
